# Impact of immune escape mutations on HIV-1 fitness in the context of the cognate transmitted/founder genome

**DOI:** 10.1186/1742-4690-9-89

**Published:** 2012-10-30

**Authors:** Hongshuo Song, Jeffrey W Pavlicek, Fangping Cai, Tanmoy Bhattacharya, Hui Li, Shilpa S Iyer, Katharine J Bar, Julie M Decker, Nilu Goonetilleke, Michael KP Liu, Anna Berg, Bhavna Hora, Mark S Drinker, Josh Eudailey, Joy Pickeral, M Anthony Moody, Guido Ferrari, Andrew McMichael, Alan S Perelson, George M Shaw, Beatrice H Hahn, Barton F Haynes, Feng Gao

**Affiliations:** 1 Duke Human Vaccine Institute, Duke University Medical Center, Durham, NC 27710, USA; 2 Department of Medicine, Duke University Medical Center, Durham, NC 27710, USA; 3 Theoretical Division, Los Alamos National Laboratory, Los Alamos, NM, 87545, USA; 4 Department of Medicine, University of Pennsylvania, Philadelphia, PA, 19104, USA; 5 Department of Microbiology, University of Pennsylvania, Philadelphia, PA, 19104, USA; 6 Department of Medicine, University of Alabama at Birmingham, Birmingham, AL, 35294, USA; 7 Weatherall Institute of Molecular Medicine, University of Oxford, Oxford, England, OX3 9DS, UK; 8 Department of Pediatrics, Duke University Medical Center, Durham, NC 27710, USA; 9 Department of Surgery, Duke University Medical Center, Durham, NC, 27710, USA; 10 Department of Immunology, Duke University Medical Center, Durham, NC, 27710, USA; 11 The Santa Fe Institute, Santa Fe, NM, 87501, USA

**Keywords:** Human immunodeficiency virus type I, Viral fitness, Cytotoxic T lymphocytes, Immune escape mutation, Transmitted/founder virus, Mathematical model

## Abstract

**Background:**

A modest change in HIV-1 fitness can have a significant impact on viral quasispecies evolution and viral pathogenesis, transmission and disease progression. To determine the impact of immune escape mutations selected by cytotoxic T lymphocytes (CTL) on viral fitness in the context of the cognate transmitted/founder (T/F) genome, we developed a new competitive fitness assay using molecular clones of T/F genomes lacking exogenous genetic markers and a highly sensitive and precise parallel allele-specific sequencing (PASS) method.

**Results:**

The T/F and mutant viruses were competed in CD4^+^ T-cell enriched cultures, relative proportions of viruses were assayed after repeated cell-free passage, and fitness costs were estimated by mathematical modeling. Naturally occurring HLA B57-restricted mutations involving the TW10 epitope in Gag and two epitopes in Tat/Rev and Env were assessed independently and together. Compensatory mutations which restored viral replication fitness were also assessed. A principal TW10 escape mutation, T242N, led to a 42% reduction in replication fitness but V247I and G248A mutations in the same epitope restored fitness to wild-type levels. No fitness difference was observed between the T/F and a naturally selected variant carrying the early CTL escape mutation (R355K) in Env and a reversion mutation in the Tat/Rev overlapping region.

**Conclusions:**

These findings reveal a broad spectrum of fitness costs to CTL escape mutations in T/F viral genomes, similar to recent findings reported for neutralizing antibody escape mutations, and highlight the extraordinary plasticity and adaptive potential of the HIV-1 genome. Analysis of T/F genomes and their evolved progeny is a powerful approach for assessing the impact of composite mutational events on viral fitness.

## Background

HIV-1 fitness plays a critical role in virus persistence, transmission, pathogenesis, and disease progression [1-9]. Because of HIV-1 error prone reverse transcriptase and rapid virus turnover and immune selection pressure, a small viral fitness change may have a significant impact on HIV-1 evolution [10]. Strong pressure from cytotoxic T lymphocyte (CTL) responses selects virus mutants, with complete replacement of CTL sensitive viruses within weeks of HIV-1 infection [11,12]. These CTL escape mutations have been widely studied for their ability to impair viral fitness [13-17]. If fitness is reduced there may be a decrease in viral load, leading to long-term HIV-1 control and decreased probability of transmission to new hosts [7,9]. Moreover, if less fit viruses are transmitted into new hosts, viral loads may be lower and a better clinical outcome expected [9], although the effect may not be sustained into chronic infection [8].

The majority of fitness studies have compared viruses *in vitro* using either parallel or competition assays [13-19]. In the latter, competition between two viruses is performed in the same culture, and the relative fitness is determined by the dynamic changes of the ratio of viruses over time [17,20,21]. The proportion of each virus in the culture is determined by detecting unique artificial markers introduced into the viral genomes or mutations by population sequencing, clone sequencing or real-time PCR [13,14,16,18,19,22,23]. However, there are several factors that may compromise evaluation of viral fitness. A lab-adapted viral backbone isolated from chronically infected individuals may not represent viruses that exist as a quasispecies viral population *in vivo*. When mutations of interest or gene fragments are introduced into unrelated viral genomes they may have confounding effects on fitness. This may be particularly important since the mutations can have disparate effects on viral fitness in different virus backbones [14,18]. Recombination between viruses can affect the interpretation of the fitness results [24,25]. The sensitivity to detect minority variants is low for sequencing based methods [16,18,19,26,27]. Finally, only two viruses are usually compared in each assay, whereas many variants compete against each other in the HIV-1-infected individuals [13,14,16-19]. Therefore, a more reliable, sensitive and reproducible assay is needed.

Combining the unique advantages of parallel allele-specific sequencing (PASS) technology [28], unmodified HIV-1 T/F genomes [29] and new mathematical modeling, we have established a new viral fitness assay to determine the impact of CTL escape mutations emerging in early HIV-1 infection on the fitness of the transmitted/founder (T/F) virus. In the current study, we determined the impact of CTL escape mutations on viral fitness in their cognate T/F viral genome. We found that although CTL escape mutants can be associated with significant fitness costs, this can be negated by emergence of compensatory mutations. In addition, the new PASS fitness assay can be used to simultaneously compare viral fitness among multiple viruses in a single assay and determine the influence of recombination on fitness comparisons.

## Results

### Fitness comparison in the single passage assay

**Figure 1:**
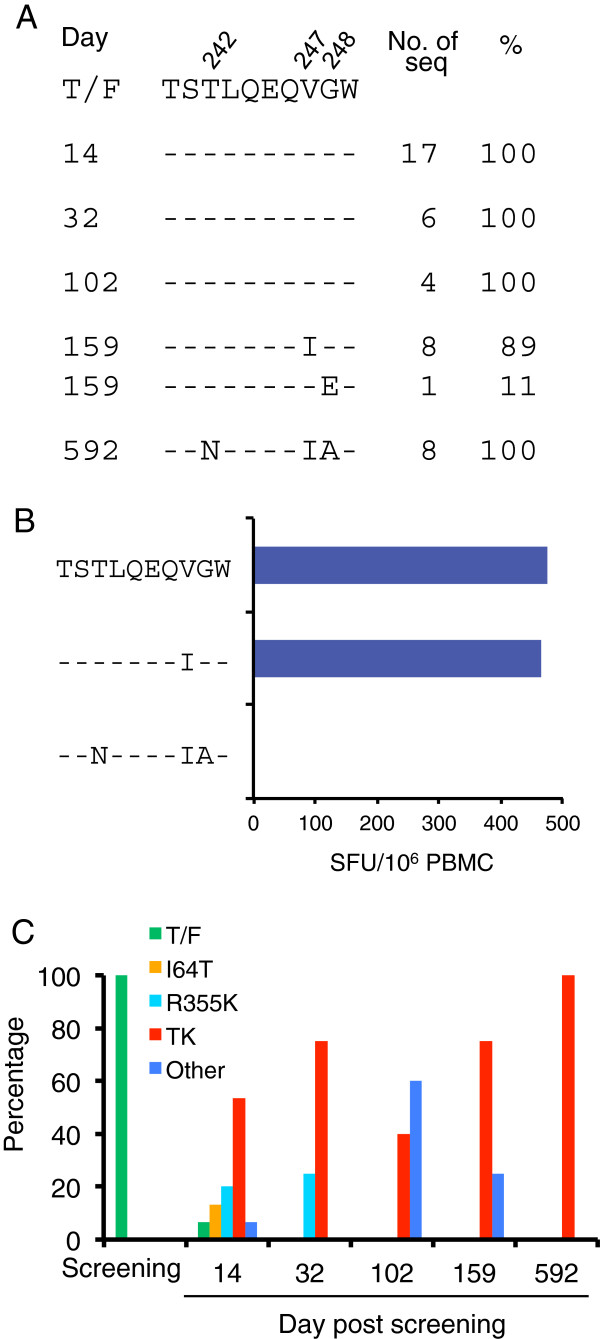
**Selection of CTL escape mutations in the HIV-1-infected subject.** (**A**) Frequencies of mutations in the TW10 CTL epitope at screening and later time points (days post screening) were determined by SGA [11,29]. The positions of amino acid substitutions are indicated above the TW10 epitope sequence. (**B**) T cell response to WT and mutant TW10 peptides (Gag_240-249_) at day 592 were determined using an ex vivo IFN-γ ELISpot assay. Positive T cell responses were defined as: ≥ 30 SFU/million and > 4 times above background. All assays were performed in duplicate. HLA typing and T cell mapping using autologous peptides spanning the transmitted founder virus was previously described [11]. (**C**) Frequencies of the viruses with I64T and/or R355K mutations at screening and later time points were determined by SGA [11,29]. The TK virus contains both I62T and R355K mutations.

**Figure 2:**
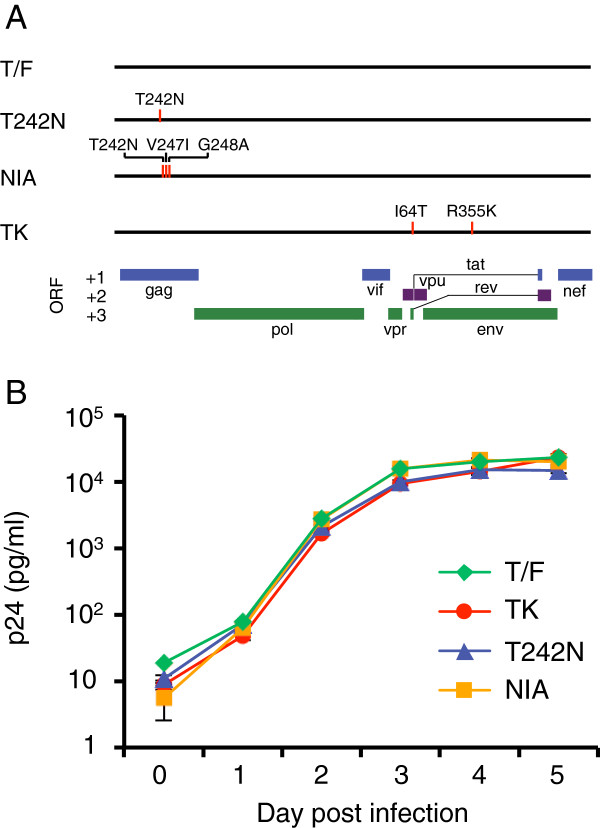
**Replication dynamics of individual viruses.** (**A**) Schematic presentation of mutation positions in the T/F virus genome. (**B**) Purified CD4^+^ T cells were infected with the same amount (5 ng p24) of CH77 T/F virus and its mutants (TK, T242N and NIA). Each virus was cultured independently in triplicates. Viral replication was monitored by measuring p24 concentrations in the cell culture supernatants. Mean values ± standard deviations are shown.

Fifteen T cell epitopes were identified in subject CH77, and CTL escape mutations were found in these epitopes in a previous study [11]. At day 592, three mutations (T242N, V247I and G248A) in the B57/5801 restricted Gag_240-249_ epitope TSTLQEQIGW (TW10) were found in all detected viral genomes (Figure 1A). The V247I mutation was detected in the majority of the viral population at day 159, but it did not impact T cell recognition (Figure 1A and 1B). However, the peptide with all three mutations resulted in complete loss of T cell recognition (Figure 1B). R355K in the epitope Env_352-369_ in Env was the earliest CTL escape mutation. It was detected only 14 days after screening (first RNA positive sample) and was present together with a reversion mutation (I64T) in the *tat/rev* overlap region in the majority of the viral population. The reversion mutation was defined as a mutation that changed back to the subtype B ancestral sequence in the absence of detectable immune selection. The virus (TK) with both R355K and I64T mutations was the predominant virus (53%) at day 14 and fixed at day 592 in the virus population (Figure 1C). To understand how those mutations affect viral fitness, we generated three infectious molecular clones (T242N, NIA and TK) by introducing mutations into the T/F viral genome (Figure 2A). T242N differed from the T/F virus by a single CTL escape mutation (T242N). NIA was different from T/F by three mutations (T242N, V247I and G248A) in the TW10 epitope. TK represented the predominant virus *in vivo* at day 14 and differed from T/F by two mutations (I64T and R355K). Purified CD4^+^ T cells were infected with these viruses individually, and all viruses had similar replication kinetics (Figure 2B).

The CTL escape mutation T242N has been reported to cause a significant fitness loss *in vitro* using the laboratory adapted NL4-3 virus backbone in several studies [14-16]. We first sought to investigate if the T242N mutation rendered the virus less fit than T/F in a single passage assay. The equal amounts of each virus were mixed to infect primary CD4^+^ T cells, and culture supernatants were harvested daily to determine the relative fitness of the compared viruses. After cDNA was made using viral RNA extracted from the cell culture supernatants, the proportion of each virus in the culture was determined by PASS. The numbers of detected viral genomes increased exponentially from day 2 to day 4, similar to the replication dynamics determined by measuring p24 concentrations (Figure 2B). We then analyzed an average of 600 (200 to 1400) viral genomes in each sample to determine the proportion of each virus in the viral population.

**Figure 3:**
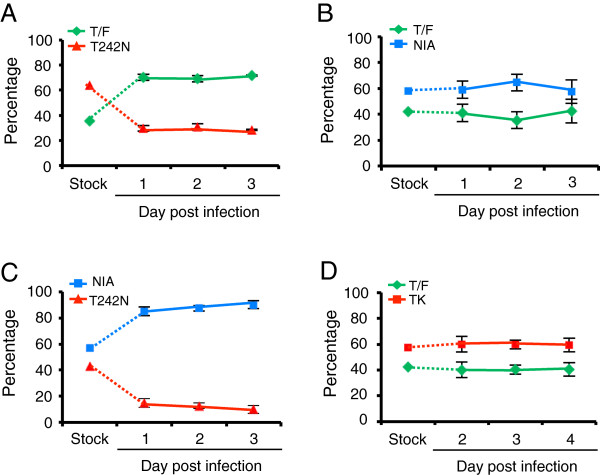
**Fitness comparison among the T/F virus and CTL escape mutants in the single passage assay.** Same amount (5 ng p24) of each virus (T/F and CTL escape mutant) was mixed to infect CD4^+^ T cells in triplicates. The viruses in the supernatant were harvested daily by completely replacing the medium. The proportion of each virus in the inoculum stock and the cell culture supernatants was determined by PASS. The relative fitness was determined by modeling the replication slope of each virus during the culture. Relative fitness was determined for (**A**) T/F versus T242N (*s*_*ij*_ = 0.009 ± 0.007), (**B**) T/F versus NIA (*s*_*ij*_ = 0.02 ± 0.02), (**C**) NIA versus T242N (*s*_*ij*_ = 0.05 ± 0.04) and (**D**) T/F versus TK viruses (*s*_*ij*_ = 0.01 ± 0.01). Similar results were obtained in two independent experiments in CD4^+^ T cells and the data from one experiment is shown. Means ± standard deviations are plotted.

When T/F and T242N were compared, T/F predominated the viral population (70%) since day 1, However, the ratio between two viruses did not change throughout the culture (Figure 3A), although the number of viral genome exponentially increased in the culture media during the same period as shown in Figure 2B. The relative fitness of T242N was similar to that of T/F (*s*_*ij*_ = -0.009 ± 0.007). We then compared T/F and NIA, which contained all three mutations in the TW10 epitope and predominated the viral population at day 592 (Figure 3B). The result showed that NIA was only 2% less fit than T/F (*s*_*ij*_ = -0.02 ± 0.02). When NIA and T242N were compared, the proportion of NIA accounted for the majority of the viral population at day 1 (85%) and slightly increased to 90% at day 3 (Figure 3C). The relative fitness of T242N was about 5% less fit than NIA (*s*_*ij*_ = -0.05 ± 0.04).

TK was the predominant virus (53%) as early as at day 14 and was fixed at day 592 in the virus population (Figure 1A), suggesting that the virus with both mutations was strongly selected *in vivo*. To determine if the CTL escape mutation in TK caused fitness loss, we compared the TK and T/F viruses. The percentages of the T/F and TK viruses (60% and 40%, respectively) did not change over time (Figure 3D). Importantly, the proportion of each virus during the culture was similar to that in the inoculum stock (57% and 43% for T/F and TK, respectively). These results showed that TK was approximately as fit as T/F (*s*_*ij*_ = 0.01 ± 0.01).

Taken together, in the single passage assay, no significant differences in relative fitness between any pair of viruses were observed, although the viruses exponentially increased in the supernatant. This is at odds with the previous observation that a virus with the CTL escape mutation T242N was less fit than the WT virus [14-16]. However, when T242N was compared to T/F or NIA, the proportion of T242N in the culture decreased by two fold compared to that in the inoculum stock (Figure 3A and 3C), suggesting that T242N was less fit than both T/F and NIA. These results indicated that the fitness differences among these viruses were not accurately measured in the single passage assay.

### Fitness comparison through multiple passages of compared viruses

Since the fitness loss caused by the T242N mutation was better revealed after multiple rounds of passages [14], and discordant fitness results have been observed between single-passage and multiple-passage fitness assays [26], we sought to investigate whether the fitness cost caused by the T242N mutation in the T/F virus can be more accurately determined by repeatedly passaging the cell-free viruses to fresh CD4^+^ T cells. Cell-free viruses harvested 3 or 4 days after infection were sequentially passaged four times to fresh CD4^+^ T cells. The proportion of each virus in the culture was determined by PASS as in the single passage assay. We analyzed an average of 636 (135 to 2197) viral genomes in each sample to determine the proportion of each virus in the samples.

**Figure 4:**
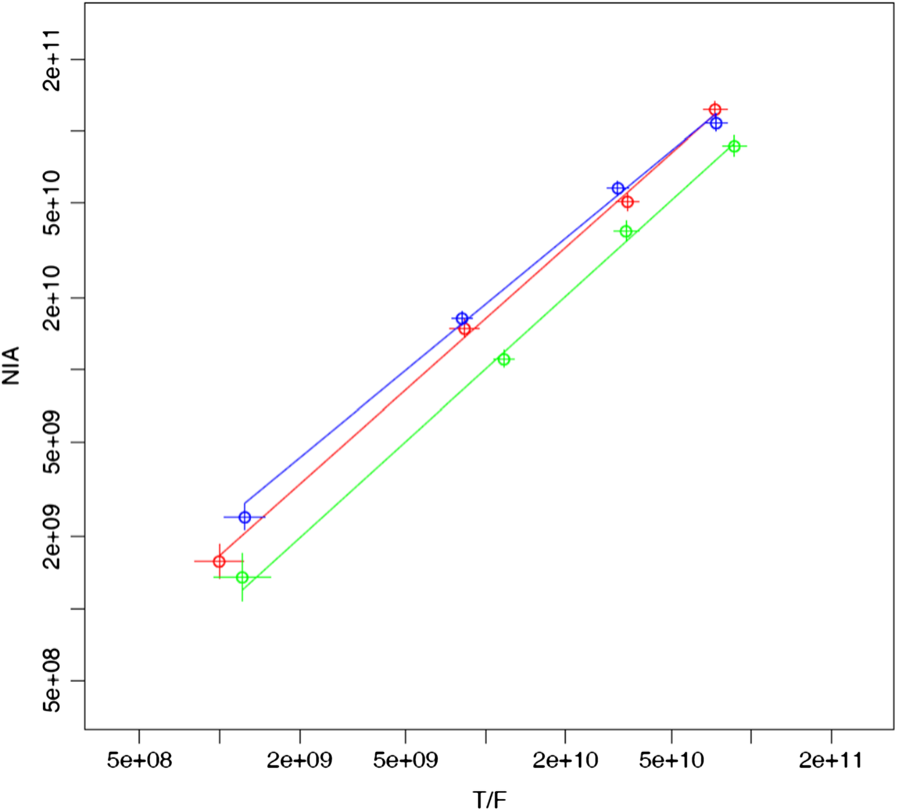
**Comparison of the growth of viruses NIA and T/F across 4 passages and three replicates.** The two axes show, on a log scale, the genomes/ml of the two viruses at the end of each growth period before passage multiplied by the dilution factor as shown in Eq. (2). The binomial sampling errors for each variant are indicated by the vertical and horizontal lines through each point. If the ratio of the average growth rates of the two viruses at each passage is a constant, the 4 points should lie on a straight line (see Methods), with the slope giving *1+s*_*ij*_. The maximum likelihood fit to this model is shown by the solid lines. The additional assumption of constant exponential growth rate for each individual variant would mean that the points are equispaced along the best fit line (see Methods)—this assumption is clearly violated by the data.

Virus concentrations in the supernatant (p24 concentration) did not exponentially increase from early passages to later passages since the virus was harvested around the peak of the p24 concentration at each passage. Thus, previous models that assume constant exponential growth could not be directly applied to the data of the passaged viruses to determine relative fitness. Therefore, we developed a new mathematical model that does not assume constant exponential growth to determine relative fitness of HIV-1 strains after multiple rounds of passages. In fitting p24 and PASS data from the multiple passage experiments, only the final p24 and virus frequency values in each growth period before passage were observed. We assumed that even if the exponential growth rate of each viral variant was time variable, say due to changes in environmental conditions, the ratio of the average growth rates of any pair of viruses was the same during each growth period. We then determined the optimal value of s_ij_ (see Eq. 2) that best fitted the measured concentrations at each passage as shown in Figure 4. This procedure allows us to avoid making the assumption that the exponential growth rates, rather than only their ratios, are constant and the same during each growth period. This assumption would lead to the predictions that the quantities log*(c d*^*p*^*)* in Eq. 2 increases linearly with the passage number, *p,* giving rise to equally spaced points along the lines in Figure 4, a prediction that we found to be violated in many of our experiments.

**Figure 5:**
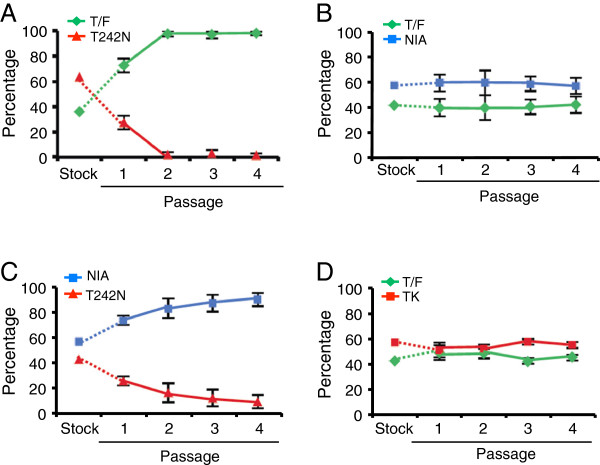
**Fitness comparison among the T/F virus and CTL escape mutants in the multiple passage assay.** Same amount (5 ng p24) of each virus (T/F and CTL escape mutant) was mixed and used to infect CD4^+^ T cells in triplicates. Cell-free viruses were harvested 3 or 4 days after infection, and 200 μl of supernatants were used to infect fresh CD4^+^ T cells. The same amounts of viruses were subsequently harvested and used to infect fresh CD4+ T cells for four passages. The proportion of each compared virus harvested from each passage was determined by PASS analysis. The relative viral fitness was estimated using the newly developed mathematical model that does not assume constant exponential growth. Relative fitness was determined for (**A**) T/F versus T242N (*s*_*ij*_ = -0.42 ± 0.03), (**B**) T242N versus NIA (*s*_*ij*_ = 0.37 ± 0.14), (**C**) T/F versus NIA (*s*_*ij*_ = -0.03 ± 0.03), and (**D**) T/F versus TK (*s*_*ij*_ = 0.05 ± 0.02). Similar results were obtained in two independent experiments and data from one experiment is shown. Means ± standard deviations are plotted.

At passage 1, T/F and T242N accounted for 73% and 27% of the viral population, respectively, although T242N was in nearly two fold excess in the inoculum (36% T/F and 64% T242N) (Figure 5A). At passage 2, T/F (98%) almost completely replaced T242N and dominated in succeeding passages. Analysis of the data using this new model showed that T242N was 42% less fit than T/F (*s*_*ij*_ = -0.42 ± 0.03). This result is similar to that reported in a previous study [14], confirming that T242N mutation could cause a significant fitness loss in its cognate T/F virus backbone or in a NL4-3 backbone. However, the number of passages required for T/F to dominate T242N was fewer than that previously observed, suggesting that the levels of fitness loss caused by the T242N mutation varies in different backbones.

When T242N and NIA, which was naturally selected *in vivo,* were compared, NIA was also more fit than T242N. During four passages, NIA continuously increased in the viral population (from 74% to 91%) while T242N was gradually outcompeted (from 26% to 8%), although the proportion of each virus in the inoculum was similar (43% T242N and 57% NIA) (Figure 5B). As a result, T242N was 37% less fit than NIA (*s*_*ij*_ = -0.37 ± 0.14). We then compared T/F and NIA and found that the proportion of each virus did not change throughout all passages (Figure 5C), indicating that the fitness of both viruses was similar (*s*_*ij*_ = -0.03 ± 0.03). Taken together, our results confirmed that the T242N mutation alone in the TW10 CTL epitope caused significant fitness loss in the multiple passage assay. However, two additional mutations in the same epitope restored the viral fitness to the wild type virus level.

We also compared the TK and T/F viruses to determine if the CTL escape mutation in TK caused fitness loss after multiple passages. The proportion of each virus was similar to that in the inoculum stock (57% and 43% for T/F and TK, respectively) throughout passages, suggesting both viruses were similarly fit (Figure 5D).

### Frequent recombination in fitness assay can be assessed by PASS

**Figure 6:**
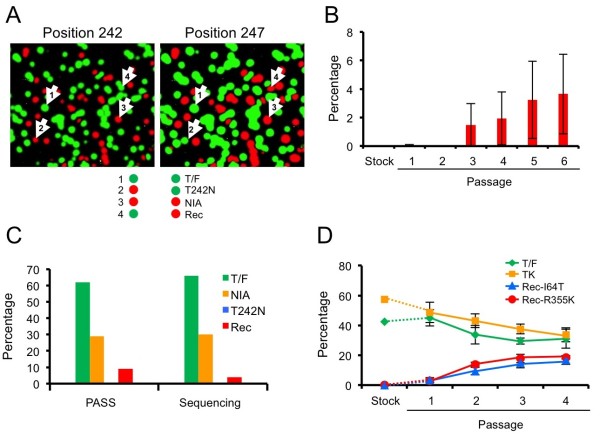
**Detection of recombinant viruses generated in the viral fitness assay.** Three viruses (T/F, T242N and NIA) were co-cultured and passaged six times. The virus in the supernatants was harvested after 3 or 4 days in each passage and subjected to PASS analysis. (**A**) The same amplicons in the PASS gel were first probed to determine the bases at position 242 and subsequently the bases at position 247. The linkage analysis of bases at both positions was performed to distinguish the three viruses and the recombinant. The T/F virus (arrow 1) has 242T (green) and 247V (green); the T242N virus (arrow 2) has 242N (red) and 247V (green); the NIA virus has (arrow 3) has 242N (red) and 247I (red); and the recombinant (rec; arrow 4) has 242T (green) and 247I (red). The result from the viruses harvested at passage 5 from one experiment is shown. (**B**) Frequency of the recombinant genomes during multiple passages. The recombinants between T/F and NIA were detected for each passage. The viral culture was carried out in triplicate. Means ± standard errors are plotted. (**C**) Comparison of frequencies of the recombinant virus and other three viruses (T/F, T242N and NIA) in the same sample determined by PASS (596 genomes) and SGA sequencing (47 genomes). (**D**) Detection of recombinant viral genomes between the T/F and TK viruses during four rounds of passages. Two recombinants (viruses with I64T or R355K mutation) were detected by linkage analysis of mutations at positions 64 in Tat and 355 in Env. The viral culture was carried out in triplicate. Means ± standard deviations are plotted.

One feature of the PASS fitness assay is that nucleotides at multiple sites in the same viral genomes can be determined, and thus recombination between compared viruses can be accessed through linkage analysis of these sites. We then sought to test how frequently recombinants were generated in the culture using the PASS assay. We infected the same CD4^+^ T cells with three viruses (T/F, T242N and NIA) and passaged the viruses six times. Since the PCR products amplified by PASS were immobilized in the acrylamide gel and could be re-probed multiple times, the linkage between multiple mutations in the same viral genomes was determined [28,30]. Thus we could easily separate the recombinant from the three parental viruses by performing linkage analysis of two nucleosides at positions 242 and 247: 242T/247V (T/F), 242N/247V (T242N), 242N/247I (NIA), and 242T/247I (recombinant) (Figure 6A). Our analysis showed that a recombinant that was not present in the virus inoculum was detected at 0.18% at passage 1 in one culture and became detectable after 3 or 4 passages in the other two cultures. It gradually increased in all three independent cultures and reached to an average of 3.6% at passage 6 (Figure 6B). The recombinant was detected as high as 9% (55 of 596 viral genomes) in one culture in which the recombinant was detected at passage 1.

**Figure 7:**
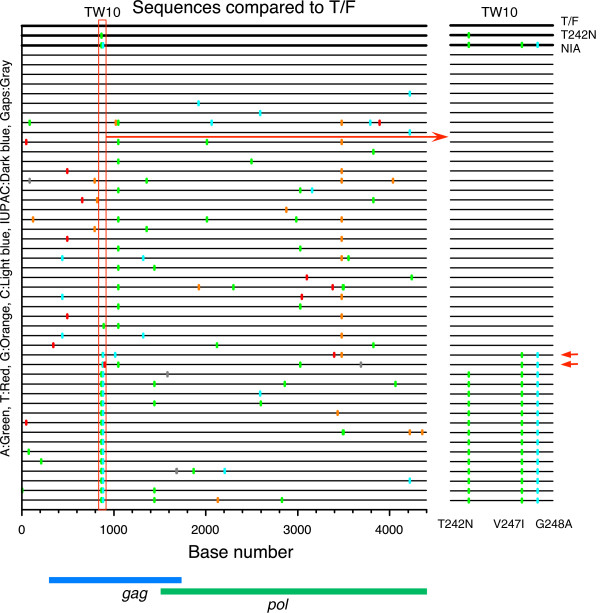
**Identification of recombinant viral genomes among compared viruses by SGA.** The 5’ half genome (4396 bp) was amplified by SGA from 47 viruses from a passage 5 culture. The Highlighter plot denotes the location of nucleotide substitutions compared to the T/F sequences, with their positions in the *gag* and *pol* genes indicated at the bottom. The three parental viruses (T/F, T242N and NIA) are indicated by thicker lines at the top. Nucleotide substitutions are color coded. The TW10 CTL epitope region (indicated by a red box) was enlarged to better show the nucleotide identities at three sites (T242N, V247I and G248A) in the viral population (right panel). Two recombinant genomes are indicated by arrows.

To confirm whether the recombinants detected by PASS accurately represented their proportion in the viral population and whether any particular mutations accumulated during *in vitro* culture affect viral fitness, we analyzed 47 5’ half HIV-1 genome sequences (4396 bp) by SGA from one passage 5 virus. Sequence analysis showed 66% T/F, 30% NIA, 4% recombinant, and no T242N (Figure 6C and Figure 7). These results were very similar to what were identified among 596 viral genomes by PASS (62% T/F, 29% NIA, 9% recombinant, and no T242N). Examination of these sequences showed that the majority of sequences (83%) had ≤3 mutations, and no sequences contained more than 7 mutations (Figure 7). The majority of mutations were random across the genome and no non-synonymous mutations in the *gag* and *pol* coding regions dominated the viral populations, suggesting that no particular genetic variants accumulated over time and hence affected fitness of the compared viruses.

**Figure 8:**
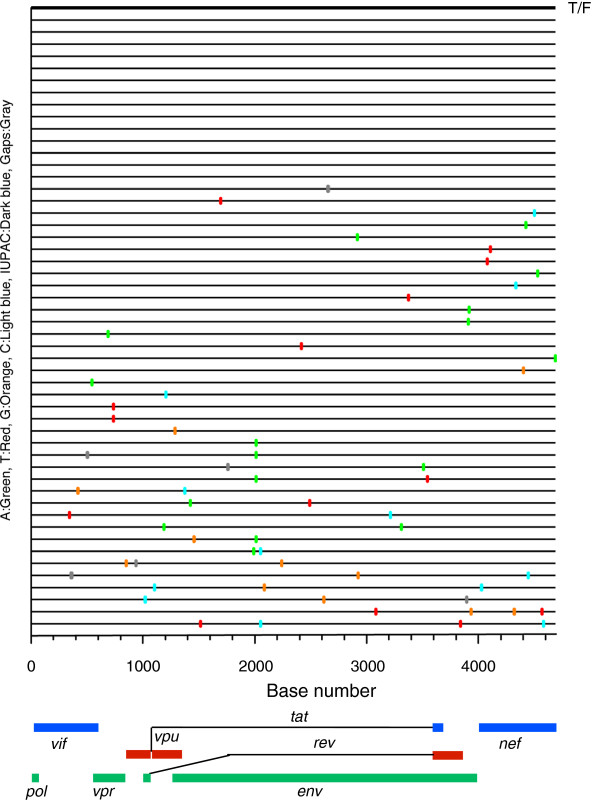
**Analysis of 3’ half HIV-1 genome sequences obtained by by SGA.** The 3’ half genome (4685 bp) was amplified by SGA from 47 viruses after five passages of the T/F virus. The Highlighter plot denotes the location of nucleotide substitutions compared to the T/F sequence, with their positions in the *vif, vpr, vpu, tat, rev, env* and *nef* genes indicated at the bottom. The T/F sequence is indicated by thicker lines at the top. Nucleotide substitutions are color coded.

Since recombination was detected between two sites that were only 13 bases apart in the T/F and NIA genomes, we then tested if a higher recombination rate occurred between viruses with different bases at a longer distance. TK was different from T/F by two mutations (I64T and R355K in Tat/Rev and Env, respectively) that were separated by 1258 bases. Using the same linkage analysis, we analyzed both mutation sites and detected both possible recombinants (virus with only the I64T or R355K mutation) in 7.1% of the viral population at passage 1 (Figure 6D). Each recombinant accounted for roughly half of the recombinant population. Both recombinants continuously increased to 35.4% at passage 4 at a similar rate (Figure 6D). These results suggested that the recombinants that gradually become predominant in the viral population in the culture were more fit than the parental viruses *in vitro*. To determine if the I64T mutation was the result of reversion mutation during the multiple passages, we analyzed 51 3’ half genome sequences obtained by SGA after 6 passages of the T/F virus. Random mutations (1-4) were detected in 37 sequences while no mutations were found in the other 14 sequences (Figure 8). Importantly, no mutations at the I64T site were detected. This strongly supports that the I64T recombinant detected in the co-culture of the T/F and TK viruses were indeed the results of recombination. Interestingly, both recombinants were detected in the individual at day 14 at low percentages and then outcompeted by the TK virus (Figure 1C). Taken together, the data demonstrate that recombination frequently occurs during the competitive fitness assay and can significantly affects the result of fitness assay.

## Discussion

A better understanding of the fitness cost of mutations associated with immune escape can have important implications for understanding viral pathogenesis, transmission and vaccine development [1-9]. To accurately determine fitness of viruses present *in vivo,* we developed a new PASS fitness assay with unmodified HIV-1 genomes that were identified *in vivo*. Using this assay, we have precisely determined the impact of mutations in the cognate T/F virus genome. Although much more effort is required to generate T/F IMCs to study viral fitness than to use existing IMCs from unrelated viruses, we have generated over 20 IMCs and identified a number of CTL cell escape mutations through analysis of longitudinal follow-up sequences [31]. They can serve as ideal candidates for further study of the impact of immune escape mutations on the fitness of cognate viral genomes. We determined the fitness cost of two CTL escape mutations (R355K in Env and T242N in Gag) in the context of other mutations in the cognate viral genomes (TK and NIA, respectively). Both CTL escape mutations were present together with other mutations that were within or outside the CTL epitopes. Interestingly, both mutant viruses were as fit as the T/F virus. The CTL escape mutation T242N alone had a significant fitness cost as previously shown by others [14-16]. It has been shown that compensatory mutations, within or outside the T cell epitopes, can partially restore the fitness loss caused by T cell escape mutations [7,14,15,32,33]. Here, we demonstrate that fitness loss by CTL escape mutations can be completely restored by the mutations in the same epitope in their cognate virus genome. The TK virus with both the I64T mutation in Tat/Rev and the R355K CTL escape mutations in Env represent a naturally selected virus *in vivo.* Although the TK virus had two mutations, it was as fit as the T/F virus. No fitness loss of the TK virus also explained why it outcompeted the T/F *in vivo* and dominated the viral population under the CTL selection pressure only 14 days after the date when the T/F virus was inferred. More importantly, this represents another case, like the NIA virus, demonstrating that the overall viral fitness is not impaired by the presence of both a CTL escape mutation and an additional mutation in the T/F viral genome.

These observations may have important implications for understanding pathogenesis and vaccine development. Many mutations that were not associated with immune responses are often identified in the viral genome and their biological functions have not been well elucidated [31]. Results from other studies and ours indicate that such mutations, especially those reversion mutations, may play an important role in compensating fitness loss caused by the CTL escape mutations, so the virus can survive the unfavorable environment in the presence of immune selection pressures. Alternatively, they may represent adaptations that favor virus replication in the host target cell environment.

In two comparison pairs (T/F verses T242N and NIA verses T242T), no difference in replication rates were observed for the compared viruses in the single passage assay even though the viruses increased exponentially in the cell culture medium. However, the fitness loss by the T242N mutation was clearly demonstrated when the cell free viruses were passaged multiple times as shown in this study and a previous report [14]. This may be explained by the following reasons. First, the T242N mutation affects the viral replication through interacting with cyclophilin A [15]. Since the viral entry was not impaired by the T242N mutation, the T242N was marginally less fit than T/F in the single passage assay. The p24 concentration of T242N was only less than two fold lower than that of the wt or T/F virus at the end of the culture when virus replication plateaued as shown in a previous study [15] and by our result (Figure 2B). Second, the infectious viruses generated during the first 2 days of culture were far fewer than those in the inoculum (90,000 infectious units (IU)/ml versus 200 IU/ml). Thus, it is unlikely that the majority of the new infections during the four-day culture were initiated by the cell-free viruses newly generated in the single passage culture. Third, cell-to-cell infection was about 100-1000 time more efficient than the cell-free virus in culture [34]. Thus, the subsequent infection is most likely established through the cell-to-cell infection in the single passage culture. If the efficiency of the cell-to-cell infection was equal for the compared viruses with the identical *env* gene, the proportion of each virus might not change over time in the culture. As a result, the relative fitness determined in the single passage culture would be similar between the compared viruses. However, by passaging the cell-free viruses to the fresh CD4^+^ T cells, the cell-free viruses would compete with each other multiple times. Since the more fit virus accounted for a larger portion of the viral population at each passage, they would infect more cells in each subsequent passage as shown in this study and a previous report [14]. Importantly, the dynamics of proportion changes for each compared virus between the inoculum and the first passage was consistent with the viral replication slope during the subsequent passage(s), suggesting that the initial changes in viral proportion from the inoculum to the first passage represented intrinsic viral fitness differences and could be further confirmed by subsequence passages (Figure 5). Since virus growth was not in exponential stage throughout passaging, previous models that assume constant exponential growth could not be used to determine relative fitness between compared viruses. Thus, we developed a new mathematical model that does not assume constant exponential growth to measure relative fitness of HIV-1 strains after multiple rounds of passages. Taken together, results from this and other studies indicate that multiple passages are needed to better determine the fitness difference between compared viruses if the proportions of viruses change obviously between the inoculum and the first passage, but no difference in replicative slope is observed during the first passage (Figure 3A and 3C). However, if the proportions of viruses do not change from the inoculum to the culture throughout the first passage, this indicates that the compared viruses are similarly fit and additional passages may not be necessary (Figure 3B and 3D). When the difference in replication slope is clearly demonstrated over time during the first passage as shown in our previous study [35], the relative fitness can be determined without further passaging.

Viral fitness is generally determined by either directly sequencing the bulk PCR products or by detecting markers that are incorporated at different locations of the viral genome [16,18,19,26,27]. Thus, the impact of recombination on viral fitness analysis during culture has not been fully elucidated. Since the high frequency recombination has been observed in culture [24,25], it is likely that recombination has a significant impact on viral fitness assays, although recombination was considered low in a previous study [27]. The PASS assay can detect multiple sites in the same viral genomes, and the linkage analysis of these sites allows detection of recombination among a limited number of mutations within a small region (≤ 2 kb) in the viral genome. The emergence and gradual increase of the recombinants to as high as 35.4% of the viral population *in vitro* suggest recombinant viruses are more fit than both parental viruses. Interestingly, all three recombinants (V247I, I64T and R355K) detected *in vitro* in this study were also identified *in vivo* (Figure 1A and 1C), suggesting that those recombinant viruses are naturally present in HIV-1-infected individuals. The V247I virus was detected as the predominant virus before the CTL escape mutation T242N was detected and then fixed together with the T242N mutation in the viral population. Since V247I was a reversion mutation, the emergence and domination of this virus suggest that it is also more fit than the T/F virus *in vivo* although this still needs to be experimentally confirmed. The accumulation of more fit viruses with V247I mutation can also readily compensate the fitness cost of the T242N mutation when it is selected later. Two other recombinants (viruses with either I64T or R355K mutation) were only detected *in vivo* at low frequencies shortly after infection and then quickly replaced by the TK virus (Figure 1C), suggesting they are less fit *in vivo* under selection pressure. This suggests that although both recombinants are more fit than the T/F and TK viruses *in vitro*, the TK virus, under the immune selection pressure, has a higher replication advantage over both of the recombinants *in vivo*.

Although it was possible that the one base difference could be caused by mutation, the odds for one mutation to occur at a particular site in different experiments at various time points was much smaller than recombination between two existing parental viruses. The point mutation rate for HIV has been estimated as ~ 2.2-5.4 × 10^-5^ per base per replication *in vitro*[36,37]. Because we were interested in mutation to a specific nucleotide, we divided the point mutation rate by 3 (0.73 -1.8 × 10^-5^), and because either of the two possible bases could mutate, the probability of the observed sequence occurring by mutation was 1.4 - 3.6 × 10^-5^. In contrast, recent estimates of the recombination rate were 1-1.4 × 10^-5^ per site per generation [38,39]. Thus if the recombination target is 13 nucleotides between T/F and NIA, the expected recombination rate is 1.8 × 10^-4^ per generation. Thus, it is about 5-12 times more likely than a point mutation to a specific nucleotide at one or the other site. In the case of the comparison between T/F and TK, the recombination target is 1258 base long and thus the recombination probability is about 1.8 × 10^-2^ per generation. This is 500-1200 times more likely to be due to the recombination than the point mutation.

The mutations detected in the recombinant genomes were the same as those selected by CTL or reversion mutations *in vivo*. There is a possibility that they represented reversion mutations during the *in vitro* culture. However, analysis of 51 SGA sequences did not show the reversion mutation (I64T) after the T/F virus was passaged six times. In addition, analysis of over 2000 viral genomes from the co-culture of the NIA and T242N viruses (both with the T242N mutation) at passage 4 did not show the wild type base at position 242. This is in good agreement with the *in vivo* observations, in which the T242N mutation reverted back to the wild type base after months of infection [8,32,40-42]. These results demonstrate that mutations detected in the recombinant genomes were the results of the recombination between parental viruses, not the results of reversion or random mutations during the time period of the assay. HIV-1 is known to be highly recombinogenic [24,25,38,39,43]. Results from this study indicate that the recombination frequently occurs between compared viruses in competition fitness assays and should be considered for accurate estimation of viral fitness. If the different nucleotides are few and within 1.5 kb in the compared viral genomes, the impact of recombination on the fitness analysis can be determined by the PASS fitness assay.

There are several advantages of the new PASS fitness assay. First, no modifications in the viral genome are needed. Second, the mutations of interest are directly measured. Third, fitness can be simultaneously compared for multiple viruses. Fourth, recombinant viruses between targeted mutations can be detected and the influence of recombinant viruses on fitness can be accurately evaluated. Fifth, it is highly sensitive for minority variants present at 0.01% - 0.1% in the population [28]. Sixth, the detection efficiency is equally efficient for compared viruses because of the identical match between the primers and all compared viral genomes. Finally, the viruses representing those *in vivo* are used to study viral fitness, and mutations evolved from the T/F virus and identified through genetic and immunological analysis are introduced back to the cognate T/F viral genome. Using this assay, we have also found that a neutralizing antibody (nAb) escape mutant identified six months after infection was less fit than the virus without the nAb escape mutation [35]. Thus, this method can be a useful tool to precisely measure the impact of the mutations on viral fitness in the cognate viral genome and if viral fitness plays an important role in viral set points, transmission and pathogenesis of HIV-1.

## Conclusions

Analyses of the impact of CTL escape mutations on viral fitness in their cognate viral genome reveal a broad spectrum of fitness costs to CTL escape mutations in T/F viral genomes, similar to recent findings reported for neutralizing antibody escape mutations [35], and highlight the extraordinary plasticity and adaptive potential of the HIV-1 genome. A number of advantages of the PASS fitness assay make it a powerful approach for assessing the impact of composite mutational events on viral fitness by analyzing T/F genomes and their evolved progeny.

## Methods

### Infectious molecular clones and viral stocks

The infectious molecular clone (IMC) for the CH77 T/F virus was chemically synthesized in a previous study [29]. The mutations were introduced into the CH77 IMC using site-directed mutagenesis kits (Stratagene Santa Clara, CA). The virus stocks were generated by transfecting the IMCs into 293T cells as previously described [44].

### Purification of CD4^+^ T cells

Peripheral blood mononuclear cells (PBMC) were obtained through leukophereses from healthy donors under clinical protocols approved by the Duke University Institutional Review Board. PBMCs were isolated using the Ficoll-Hypaque density gradients and lymphocytes were isolated by elutriation using standard techniques. CD4^+^ T cells were negatively selected from PBMCs or lymphocytes on an autoMACS Pro Separator using the CD4^+^ T cell Isolation Kit II (Miltenyi Biotec, Auburn, CA). Purity of the CD4^+^ T cells was verified by staining with: CD16 (FITC), CD14 (PE), CD56 (PE-Cy5), CD4 (PE-Cy7), CD8 (APC), CD3 (AF700), CD19 (APC-Cy7) and CD45 (PacificBlue) and analyzed on an LSR II (BD Bioscience, San Diego, CA). All CD4^+^ T cell preparations were ≥ 95% positive for both CD3 and CD4. Purified CD4^+^ T cells were cryopreserved for later use.

### Competitive virus culture

Cryopreserved CD4^+^ T cells were thawed and stimulated for 3 days in RPMI1640 containing 10% fetal bovine serum (FBS), interleukin 2 (IL-2) (32 IU/ml; Advanced Biotechnologies, Columbia, MD), soluble anti-CD3 (0.2 μg/ml; eBioscience, San Diego, CA) and anti-CD28 (0.2 μg/ml; BD Bioscience, San Diego, CA). After stimulation, 50 μl of cell suspension (1×10^6^ cells) was seeded into each well of a 96-well plate, and infected with the virus mixture stock containing two or more viruses (5 ng p24 of each virus). Since the p24 concentrations and the TCID_50_ titers were similar among all viruses stocks, equal number of m.o.i. (~0.0003) was used for each virus. After absorption at 37°C for 4 hours, the cells were washed 3 times with RPMI 1640. The infected cells were cultured in a 24-well plate with 600 μl of RPMI 1640 containing 10% FBS and IL-2 (32 IU/ml). In the single passage assay, the culture supernatant was harvested daily and replaced with fresh medium. The virus replication kinetics was monitored by determining the p24 concentration in the supernatant using the p24 ELISA kit (PerkinElmer, Waltham, MA). Multiple-passage infection was performed by passaging the viruses repeatedly to fresh CD4^+^ T cells. The first round of infection was done as in the single-passage infection described above. The supernatant was harvested at day 3 or day 4 at the peak of the p24 production, and 200 μl of the supernatant was used to infect fresh CD4^+^ T cells (about 10 ng p24 per 10^6^ cells). The viral replication at each passage was monitored by measuring the p24 concentration. All infections were performed in triplicate.

In the competitive fitness assay, the relative fitness is determined by measuring the replication slope of each virus in the culture over time. One advantage of the competitive fitness assay is that the variation of the input of each virus does not influence the results. For example, the relative fitness determined using a higher input of the less fit virus (90%) and a lower input of more fit virus (10%) is similar to those determined using the equal input of both compared viruses (50% for each). Since the use of equal amount of each input virus could better show the replication slopes of the compared viruses, all fitness assays were carried out by using equal amount of p24 of compared viruses in this study.

### Viral RNA extraction and cDNA synthesis

Viral RNA was extracted from 50 to 200 μl of culture supernatant using the PureLink Viral RNA/DNA Mini Kit (Invitrogen, Carlsbad, CA). RNA was eluted into 20 μl of RNase free water. The viral RNA (17 μl) was used for cDNA synthesis using SuperScript III reverse transcriptase (Invitrogen, Carlsbad, CA) with the primer A4-lower: 5’-GAGTAAATTAGCCCTTCCAGTCC-3’ (nt 9082-9104, HXB2) for the *tat/env* amplicon and the primer A1-lower: 5'-CACAGGAACAAGCAGCCAGGTC-3 (nt 1152-1173) for the *gag* amplicon. The cDNA was either immediately used for the PASS assay or stored at -20°C for later use.

### Determination of percentages of each virus in the culture by PASS

The PASS assay was performed as described previously [28,30]. In brief, 20 μl of acrylamide gel mix (4%) containing 1 μM acrydite-modified primer, 0.3% diallyltartramide, 5% Rhinohide polyacrylamide gel strengthener, 0.2% bovine serum albumin (BSA), 0.1% ammonium persulfate (APS), 0.1% TEMED (*NNN*’,*N*’-tetramethylethylenediamine) and cDNA template (diluted in H_2_O to a final volume of 17 μl) was casted onto a glass slide which had been treated with bind-silane (Amersham Biosciences, Piscataway, NJ). The PCR reaction mix containing 1 μM primer, 0.1% Tween-20, 0.2% BSA, 1x PCR buffer, 230 μM dNTP mix, 3.5 units of Jumpstart Taq DNA polymerase (Sigma, St. Louis, MO), and H_2_O (up to 300 μl) was added onto the gel. After sealing with a SecureSeal chamber (Grace Bio-Labs, Bend, OR), the in-gel PCR reaction was performed in a PTC-200 Thermal Cycler under the following condition: 94°C for 3 min; 65 cycles of 94°C for 30 sec, 60°C for 45 sec, and 72°C for 1 min; 72°C for 3 min. The *tat/env* fragment was amplified by using the PCR primers R-lower: 5' Acry-GGAAGCACCCAGGAAGTCAGC-3' (nt 5862-5882) and R-upper: 5'-GTATCCTCTGATGGGAGGGGCATA-3' (nt 7527-7550), and the amplicons were annealed with the sequencing primer Rev7: 5'-ATGCTACTTACTGCTTTGGTAGAGGCGCTTGATTA-3' (nt 6022-6056) to detect the I64T mutation or the sequencing primer Rev13: 5'-CCTCCTGAGGAATGGTTAAAGACTAT-3' (nt 7299-7324) to detect the R355K mutation. The *gag* amplicon was amplified by the primers A1-lower: 5' Acry-AGGGGTCGTTGCCAAAGAGTGA-3' (nt 2260-2281) and A1-upper: 5'-CACAGGAACAAGCAGCCAGGTC-3', and the amplicons were annealed with the sequencing primer C1548A: 5'-AAGGGGAAGTGATATAGCAGGATCTACTAGTA-3' (nt 1482-1513) to detect the T242N mutation or G1562A: 5'-TATAGCAGGATCTACTAGTACCCTTCAGGAACAA-3' (nt 1494-1527) to detect the V247I mutation.

After PCR amplification, single-base extension (SBE) was performed with wild type (WT) and mutant bases distinctively labeled with Cy3 and Cy5, respectively, using the primers that annealed immediately upstream of the mutation position to distinguish two compared viruses. When three viruses were compared in the same culture, the gel was re-probed again using an additional sequencing primer. The gel images were acquired using a GenePix 4000B Microarray Scanner (Molecular Devices, Sunnyvale, CA).

The two channel images (Cy5 for the WT base and Cy3 for the mutant base) were first cropped with Picture Window Pro3.5 (Digital Light & Color, Belmont, MA) to remove the edge area containing no specific signals. The cropped images were then analyzed with the Progenesis PG200 software (Nonlinear Dynamics, Durham, NC). After background subtraction, normalization, and spot filter setting, only unambiguous spots at both channels were included for further analysis. The normalized pixel count data at two mutation sites at each spot were exported into an Excel file with a unique identifier. By comparing each spot’s normalized values at both channels, the different viruses were identified based on the base identity, and the percentage of each compared virus in the viral population was then determined. The linkage pattern of two mutations on each viral genome was determined using the Linksys program developed in-house using macros in Excel as previous described [30]. An average of 600 (200 to 1400) viral genomes were analyzed for each sample.

The PASS error rates with DNA and RNA templates were determined in our previous study [45]. Its error rate with the HIV-1 RNA template is 5.5 × 10^-5^, which is far below the frequencies of the minority virus variants detected in the fitness assay. All PASS PCR and sequencing primers were tested for their specificity using plasmid DNA and viral RNA. Unexpected bases above the error rate were not observed. Thus, the PASS fitness assay was not likely to be affected by false unexpected mutations. Random mutations were generated during 65 cycles of PCR reaction. However, since all the PCR products amplified from a single viral cDNA molecule were sequenced together as a population, these random mutations, which only accounted for a very small portion of the total population, would not be detected.

### Relative fitness

The simplest analysis of fitness assumes a competition experiment during which each variant is in a phase of exponential growth. In this situation, the concentration of the various forms, *c*_*i,*_ grows with time, *t,* as *c*_*i*_*= c*_*i*_^*0*^ exp(*k*_*i*_*t)*, where *k*_*i*_ is the Malthusian growth parameter of this variant and *c*_*i*_^*0*^ is its concentration at time 0. In population genetics, the relative fitness, *r*_*ij,*_ of variant *i* with respect to variant *j* is often expressed as the difference *k*_*i*_*- k*_*j*_[46]. In many experimental situations, however, factors extrinsic to the individual genotype influence the overall growth rate strongly. To account for this, some authors have chosen to normalize the relative fitness by the growth rate of one of the variants [17], so that this normalized relative fitness is given as *s*_*ij*_*= (k*_*i*_*– k*_*j*_*)/k*_*j*_*= (k*_*i*_*/k*_*j*_*) - 1.*

The PASS assay measures the relative numbers of various genomes in the culture volume, and we assume these are drawn from a multinomial distribution with probabilities given by the relative concentrations. The fitness difference per day is then easily estimated by fitting the function to the data:

(1) $$\text{log}\left(c_{i}/c_{j}\right)=\left(k_{i}–k_{j}\right)\;t+constant$$

To find the growth rate *k*_*j*_ needed to normalize this, the measured p24 concentration is apportioned among the various forms in the ratio observed in the PASS assay, and the result fit to an exponential function of time.

In almost all the passage experiments, and some of the replicates of the single-passage culture experiments, the assumption of constant exponential growth is seen to fail. For example, the exponential growth rate *k*_*i*_ may change with time or passage number, possibly due to changes in environmental conditions. To analyze these experiments, we assume that even under these variable growth conditions where the exponential growth rate is not constant, the normalized relative fitness *s*_*ij*_ is approximately constant over time since the competing strains experience the same environment. Under these conditions, one can show that at all times during the growth, the concentrations of the various forms should obey the equation

(2) $$\text{log}\left(c_{i}d^{p}\right)=\left(1+s_{\mathrm{ij}}\right)\text{log}\left(c_{j}d^{p}\right)+const\text{ant}$$

where *c*_*i*_ and *c*_*j*_ are the concentrations of the viral variants *i* and *j* at the *p*^th^ passage, and *d* is the factor by which the viruses at the end of one passage are diluted with fresh medium to start the next passage.

To see this, consider how the values *c*_*i*_ and *c*_*j*_ at the end of the *p*^th^ passage are related to the values *c*_*i*_^*’*^ and *c*_*j*_^*’*^ at the end of the previous passage. Since every passage begins by diluting the culture medium by a factor *d*, the starting concentrations of the two forms at the *p*^th^ passage are given by *c*_*i*_^*’*^*/d* and *c*_*j*_^*’*^*/d* respectively. If the average growth rate of the *j*^th^ form in this passage is *k*_*j*_*,* we have log [*c*_*j*_*/(c*_*j*_^*’*^*/d)*] *= k*_*j*_*t,* where *t* is the duration of the passage. From this, we obtain the relation log (*c*_*j*_*d*^*p*^*) –* log (*c*_*j*_^*’*^*d*^*p-1*^*) = k*_*j*_*t,* and a similar relation holds for the *i*^th^ virus. Taking the ratio of these two relations, one obtains the finite difference equation Δ log (*c*_*i*_*d*^*p*^*) = (1 + s*_*ij*_*)* Δ log (*c*_*j*_*d*^*p*^*),* where Δ represents the increase in a quantity during the *p*^th^ passage. Equation (2) is the unique solution to this finite difference equation if we assume *s*_*ij*_ to be the same at each passage.

To fit to this equation to the data, we need the absolute concentrations of the various genomes, whereas the PASS assay is only standardized to provide the relative counts accurately. We therefore use the p24 assay to obtain the total concentration of virions in the culture medium. We have used an estimate of 40000 genomes/pg of p24 [47] to determine the HIV-1 concentration, but the numerical estimates of the normalized selection coefficients is independent of the value of this constant. We obtain the estimate of *s*_*ij*_ by numerically maximizing the Poisson likelihood of the observed PASS counts when the logarithms of the underlying concentrations are restricted to the linear form given by Eq. (2). In those cases where we do observe constant exponential growth, the estimates obtained by this method agree with those from fitting Eq. (1) to the data.

## Abbreviations

CTL: Cytotoxic T lymphocyte; T/F virus: Transmitted/founder virus; PASS: Parallel allele-specific sequencing; SGA: Single genome amplification; PBMC: Peripheral blood mononuclear cells; nAb: Neutralizing antibody; IMC: Infectious molecular clone; FBS: Fetal bovine serum; IL-2: Interleukin 2; m.o.i: Multiplicity of infection; BSA: Bovine serum albumin; APS: Ammonium persulfate; TEMED: *N*,*N*,*N*’,*N*’-tetramethylethylenediamine; SBE: Single-base extension.

## Competing interests

The authors declare that they have no competing interests.

## Authors’ contributions

FG, HS, JWP, NG, AM, GMS, BHH, BFH, conceived the study and wrote the manuscript. TB and ASP developed mathematical models, determined relative fitness and wrote the manuscript. HS, JWP, FC, NG, MKPL, AB, BH, MSD, JE, JP, MAM and GF acquired experimental data and involved in data analysis. HL, SSI, KJB and JMD generated infectious molecular clones. All authors have given the final approval of the manuscript.
